# High dose lansoprazole combined with metronomic chemotherapy: a phase I/II study in companion animals with spontaneously occurring tumors

**DOI:** 10.1186/s12967-014-0225-y

**Published:** 2014-08-21

**Authors:** Enrico P Spugnini, Sabrina Buglioni, Francesca Carocci, Menicagli Francesco, Bruno Vincenzi, Maurizio Fanciulli, Stefano Fais

**Affiliations:** SAFU Department, Regina Elena Cancer Institute, Rome, Italy; Ambulatorio Veterinario “Le Accademie”, Rome, Italy; Centro Veterinario Gianicolense, Rome, Italy; Università Campus Biomedico, Rome, Italy; Department of Drug Research and Medicine Evaluation, National Institute of Health (ISS), Anti-Tumor Drug Section, Rome, Italy

**Keywords:** Chlorambucil, Cyclophosphamide, Lansoprazole, Piroxicam, Proton pump, Water alkalizer

## Abstract

**Background:**

The treatment of human cancer has been seriously hampered for decades by resistance to chemotherapeutic drugs. A very efficient mechanism of tumor resistance to drugs is the proton pumps-mediated acidification of tumor microenvironment. Metronomic chemotherapy has shown efficacy in adjuvant fashion as well as in the treatment of pets with advanced disease. Moreover, we have shown in veterinary clinical settings that pre-treatment with proton-pumps inhibitors (PPI) increases tumor responsiveness to chemotherapeutics. In this study pet with spontaneously occurring cancer have been recruited to be treated by a combination of metronomic chemotherapy and high dose PPIs and their responses have been matched to those of a historical control of ten patients treated with metronomic chemotherapy alone.

**Methods:**

Single arm, non randomized phase II open study, with historical control group, evaluating safety and efficacy of the combination of metronomic chemotherapy and alkalization. Twenty-four companion animals (22 dogs and 2 cats) were treated adding to their metronomic chemotherapy protocol the pump inhibitor lansoprazole at high dose, and a water alkalizer. Their responses have been evaluated by clinical and instrumental evaluation and matched to those of the control group.

**Results:**

The protocol was overall well tolerated, with only two dogs experiencing side effects due to gastric hypochlorhydria consisting with vomiting and or diarrhea. In terms of overall response, in the alkalized cohort, 18 out of 24 had partial or complete responses (75%), two patients had a stable disease and the remaining patients experienced no response or progressive disease. On the other hand, only one patient in the control group experienced a complete response (10%) and three other experienced short lived responses. Median time to terminal event was 34 weeks for the experimental group versus 2 weeks in the controls (p= 0.042).

**Conclusions:**

Patient alkalization has shown to be well tolerated and to increase tumor response to metronomic chemotherapy as well the quality of life in pets with advanced cancer. Further studies are warranted to assess the efficacy of this strategy in patients with advanced cancers in companion animals as well as in humans.

## Introduction

Cancer initiation, invasion and dissemination are dynamic phenomena influenced by tumor microenvironment and host factors. Standard anticancer drugs were devised accordingly to Erlich’s magic bullet concept and rely on their capacity to directly kill malignant cells. In the attempt to maximize their efficacy, they are administered at a maximal tolerated dose (MTD), the highest amount of the drug associated with tolerable toxicity and manageable side effects [1,2]. As a consequence of this approach, patients must go through long periods off therapy, allowing for their full recovery from the adverse effects of chemotherapy. These interruptions, however, also permit cancer cells to become resistant to chemotherapy and hence to promote a disease recurrence [3,4]. As a consequence, despite the discovery of a significant number of these drugs and the huge number of clinical trials that have been undertaken to develop novel multi-drugs protocols, results has been modest in terms of cure or life extension of cancer patients, especially those with advanced-stage or metastatic disease [5]. A new therapeutic paradigm has recently been devised, consisting of the use of low-dose chemotherapeutics at short intervals (so-called, metronomic chemotherapy), in the absence of extended drug-free periods [6,7]. Metronomic chemotherapy is not only almost devoid of toxic effects, but also exerts e direct citotoxicity combined with an antiangiogenic activity resulting in significant efficacy [8,9]. Several studies in human and veterinary oncology have shown efficacy of low dose alkylating agents such as cyclophosphamide and chlorambucil to directly approach refractory cancers or as adjuvant therapy for highly metastatic or metastasizing tumor [10-17]. The other factor to be taken into account when planning a treatment procotol, is the response to chemotherapy by tumor cells. This depends on the concentration of cytostatics accumulated within the cells, phenomenon that is dependent on functional expression of efflux transporters, but also on the pH of extracellular microenvironment. In fact, the acidity of tumor microenvironment is a key factor in the low level of responsiveness of tumor cells to chemotherapeutics, and proton exchangers have a crucial impact in extracellular acidification of cancer cells [18,19]. Tumor cells rely on H^+^ exchangers, in particular the vacuolar H^+^-ATPases (V-ATPases) to dispose of the dangerous protons byproduct of cancer metabolism [20-22]. The acidic tumor environment is a consequence of anaerobic glucose metabolism resulting in accumulation of acids such as lactates, leading to enhanced transmembrane pH regulation [22]. These proton pumps, together with other ion exchangers, play a crucial function in the establishment and maintenance of cancer microenvironment and their action results in the selection of more aggressive cell phenotypes able to survive in this highly hostile microenvironment, with a key role in the establishment and maintenance of chemoresistance [19]. There are several proposed mechanisms involved in this phenomenon, including decreased uptake or neutralization of weakly basic drugs by the acidic tumor microenvironment or the confinement of chemotherapy drugs within lysosomal vesicles [23]. An accelerated turnover of acidic vesicles may represent an additional tumor strategy of drug resistance based on counteracting current transportation [24]. Recently, a role of nanovesicles (exosomes) released by resistant cancer cells has been in elimination of tumor drug such as cisplatin, and extracellular acidity and exosomes release belong to a framework exerting a central role of malignant cancer unresponsiveness to chemotherapy [25]. Interestingly, the expression of proton pumps is increased in chemoresistant phenotypes and further increased by anticancer drugs as well [24]. Proton pump inhibitors have been shown to be highly effective in inhibiting V-ATPases in vitro and well tolerated and effective in murine models, improving response to chemotherapy and tumor control [24]. Moreover, in a previous study, our group showed that high dose of the proton pump inhibitor lansoprazole could reverse chemoresistance in a cohort of pets affected by spontaneous chemoresistant neoplasms, obtaining an high percentage of responders with minimal toxicities [26]. A recent clinical study in osteosarcoma patients has shown a clear effectiveness of PPI in increasing the effectiveness of standard chemotherapy, particularly in those patients that show low level of responsiveness to the standard protocols [27]. Thus, the ensemble of these studies, but also the evidence that a systemic buffering approach may represent a useful new strategy in both preventing [28] and in treating [29] cancer, suggested that a therapy combining different anti-acidic approaches, may represent a real new path in the war against cancer. Thus, the aim of this study was to investigate the feasibility, tolerability and efficacy of high dose proton pump inhibitor lansoprazole combined with water alkalization within a metronomic protocol in companion animals affected by advanced or highly metastatic neoplasms.

## Methods

### Patient selection

Privately owned canine and feline patients with advanced or highly metastatic neoplasms were selected for the study. Due to the advanced stage of the disease and the poor response to standard chemotherapy, the owners were offered two options: a) palliative therapy b) metronomic chemotherapy with the addition of high dose lansoprazole and water alkalization. Their responses have been matched against those of an historical group of 10 pets treated with metronomic chemotherapy alone.

Previous informed consent was obtained from the owners. In order to be enrolled in the study, according to the Italian law (116/92) and the guidelines defined by the ethical committee of the National Cancer Institute “Regina Elena” of Rome, Italy, patients, staged according to the World Health Organization (WHO) grading system, were considered eligible if they fulfilled the following criteria:

1. Normal renal function (normal serum blood urea nitrogen [BUN], creatinine, phosphorus, and urine specific gravity).
2. Absence of underlying life threatening diseases or other medical complications (e.g. diabetes mellitus).
3. Compliance of the owner for follow-up rechecks.
4. A presumptive life expectancy of at least four weeks.
5. Overall performance status assessed according to the modified Karnowsky system, had to be less than 3 (Table 1).

**Table 1:** **Modified Kamofsky’s performance criteria**

**Grade**	**Criteria**
0	Fully active, performs at predisease level
1	Activity less than predisease level; able to function as acceptable pet
2	Severely compromised activity; ambulatory only to point of eating,
	sleeping, and consistently eliminating in acceptable areas.
3	Completely disabled; must be force fed; unable to defecate or urinate
	in acceptable areas
4	Dead

Staging process included a thorough anamnesis, physical examination, caliper or ultrasonographic measurement of the neoplasm, complete blood cell count (CBC), serum biochemistry profile, thoracic radiographs (three projections: two laterals and one ventro-dorsal), and abdominal ultrasonography. In order to confirm the diagnoses, histological re-examination of the biopsies were performed following standard protocols, using Hematoxylin/Eosin and Hematoxylin/Van Gieson.

### Treatment

#### Experimental approach

Dogs and cats with advanced or highly metastaic spontaneous neoplasms or with chemoresistant tumors were treated with lansoprazole at the dose of 5 mg/kg from Monday through Wednesday and 1 mg/kg from Thursday to Sunday, combined with metronomic chemotherapy and a commercially available water alkalizer (alka water) added to mineral waters having pH between 7.8 and 8.0 to bring the final water pH to 9. Metronomic chemotherapy consisted with daily cyclophosphamide at the dose of 12.5 mg/m^2^, and piroxicam at the dose of 0.3 mg/kg and was the same for the experimental cohort and the historical controls [12]. Cats were treated with chlorambucil at the dose of 4 mg/m^2^ and piroxicam at the dose of 0.3 mg/kg EOD due to metabolic differences between the two species and the easier administration of chlorambucil to cats. At presentation patients were valuated accordingly to a modified Karnofsky performance scale (Table 1). Toxicity was defined as disease processes that occurred secondary to therapy and accordingly scored (Table 2). Gastro-intestinal toxicity was scored accordingly to the Veterinary Comparative Oncology Group guidelines [26,30]. In order to have the best assessment of therapy toxicoses, after every therapy owners were sent home with a questionnaire to be completed in order to record possible gastrointestinal side effects of the protocol (Table 3). Response to treatment was assessed on the basis of clinical evaluation and confirmatory biopsies. Response to treatment in terms of toxicity and tumor response were assessed prior each therapy. At that time a physical exam and tumor measure were performed. Moreover thoracic radiographs and abdominal ultrasonography were performed every two months to check for tumor spread. Tumor response was defined as follows:

- **Complete Remission** (CR) - the disappearance of all evidence of cancer in all sites for a defined period of time.
- **Partial Remission** (PR) - the decrease in size of all tumors by 50% or greater as measured by the sum of the product of two diameters of each tumor for a defined period of time.
- **Stable Disease** (SD) - the decrease of <50% or an increase of < 25% in the sum of the product of two diameters for a defined period of time.
- **Progressive Disease** (PD) - the increase of 25% or more in the sum of the product of two diameters for a defined period of time.
- **No evidence of disease** – absence of tumor growth (local recurrence or distant metastases) after surgery for highly metastatic tumors, following PPI and chemotherapy for a defined period of time.

**Table 2:** **Modified Eastern cooperative oncology group evaluation**

**Toxicity/Grade signs**	**Duration**
Hospitalization	Days
0	0
1	1
2	2–3
3	4–5
4	≥5
Neutropenia	
0	≤500 neutrophils/mL
1	1,500–2,500 neutrophils/mL
2	≥2,500 neutrophils/mL
3	500–999 neutrophils/mL
4	1,000–1,499 neutrophils/mL
Anorexia	
0	None
1	Inappetance
2	Anorexia ≤3 days duration
3	Anorexia >3 days but <5 days
4	Anorexia ≥5 days 10% weight loss
Vomiting	
0	None
1	Nausea
2	Sporadic, self-limiting
3	1–5 episodes per day, <2 days
4	6–10 episodes per day, hospitalized
Diarrhea	
0	None
1	Soft stools, responds to dietary modification
2	1–4 watery stools per day, <2 days
3	4–7 watery stools per day or >2 days
4	>7 watery stools per day or bloody,hospitalized
Infection	
0	None
1	No medication
2	Required medication
3	Debilitating
4	Threatening

**Table 3:** **Daily evaluation form sent home and made out by the owners**

**Vomiting**				
None	3 episodes per day	5 episodes per day	>5 episodes per day	>5 per day OR
	OR	OR	OR	days lasting >4 days and
	vomiting lasting 2 days	vomiting lasting 4 days	for >4 days	life threatening
**Diarrhea**				
None	2 more stools	6 more stools	>6 more stools	>6 and life
	than normal	than normal	than normal	hospitalized
**Nausea**				
None	Appetite loss with	Salivating or lip	Salivation or lip	Salivation/lip
	normal eating habits	smacking for 12 hrs	smacking for 24 hrs	smacking >24 h
**Appetite**				
Normal	With treats or diet	Appetite loss for 3 days OR	Appetite loss for 5 days OR	Loss >5 days
	change, ate 100%	With treats or diet	With treats or diet	OR
		change, ate 50% of normal	change, ate few bites	No interest,
				no appetite
**Flatulence**				
Normal	1-2 episodes per day	2-4 episodes per day	4-6 episodes per day	> 6 episodes per day
**Activity**				
Normal	Mild lethargy	Moderate lethargy, difficulty	Severe lethargy, only	Unable to
		with daily activities	gets up to go outside	rise on own

### Statistical analysis

Response to treatment was assessed using the median time to terminal event and its 95% confidence interval. The terminal event was tumor progression, recurrence or death attributable to cancer or other non-cancer causes. Time to recurrence was defined as time from the observation of tumor disappearance and estimated according to the Kaplan-Meier method [31]. The statistical significance of the differences in survival distribution among the treatment groups (experimental versus control) was evaluated by the log-rank test [32]. P values <0.05 were regarded as significant in two-tailed tests. SPSS software (version 10.00 SPSS Chicago) was used for statistical analysis.

### Evaluation of patients

Finally, the owners were questioned prior to each therapy on the activity level, performance status and food and water consumption of their animals. Patients had a complete hematological and biochemical analysis performed every two weeks, while thoracic radiographs and ultrasonograpic exam were scheduled to be performed at 1, 3, 5, 7, 9, 12, 18 months, together with a physical evaluation. Moreover, owner have been interviewed regarding their degree of satisfaction regarding the treatments. This was made as a surrogate for the psychological aspects of chemotherapy in human patients as well as to assess quality of life during the treatment. Indeed, while Treatment were scheduled to be continued until a complete remission or an absence of disease was observed for one year, a maintenance schedule was devised for the long term responders. At that time chemotherapy was discontinued while therapy with lansoprazole and patient alkalization were continued as a maintenance protocol.

## Results

**Figure 1:**
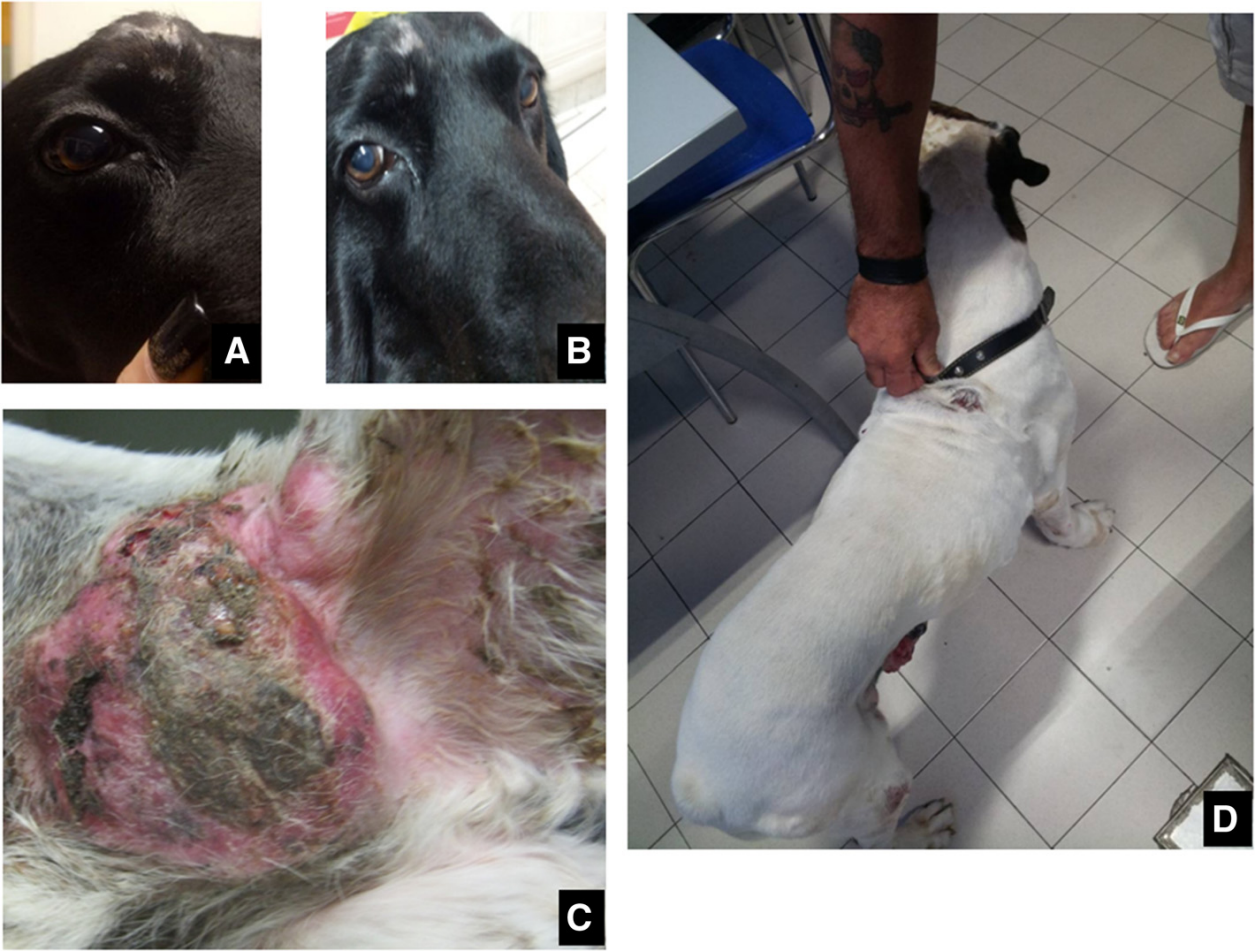
**A canine patient with a nasal sarcoma at presentation (A) and after 4 months of therapy (B), the dog had a nasal sinus sarcoma that underwent PR resulting in cessation of nasal discharge and bleeding as well as pawing at the lesion.** Another patient with a large ulcerated high grade mammary carcinoma **(C)** experiencing a long lasting PR **(D)**.

**Figure 2:**
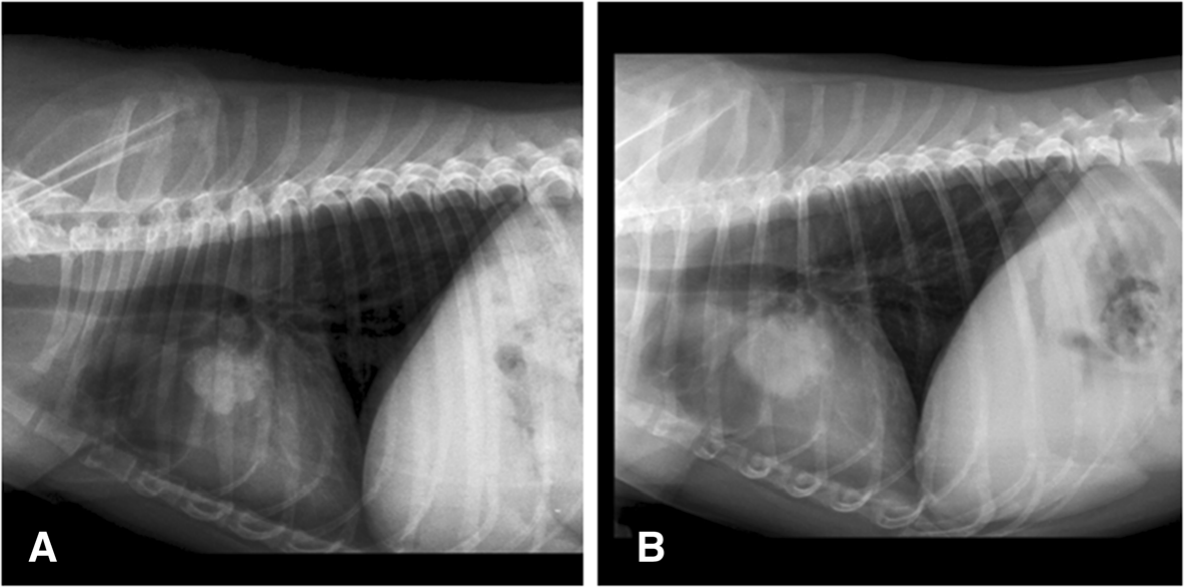
**A canine patient with lung cancer treated with metronomic chemotherapy and alkalization at presentation (A) and at four months control (B).**

**Figure 3:**
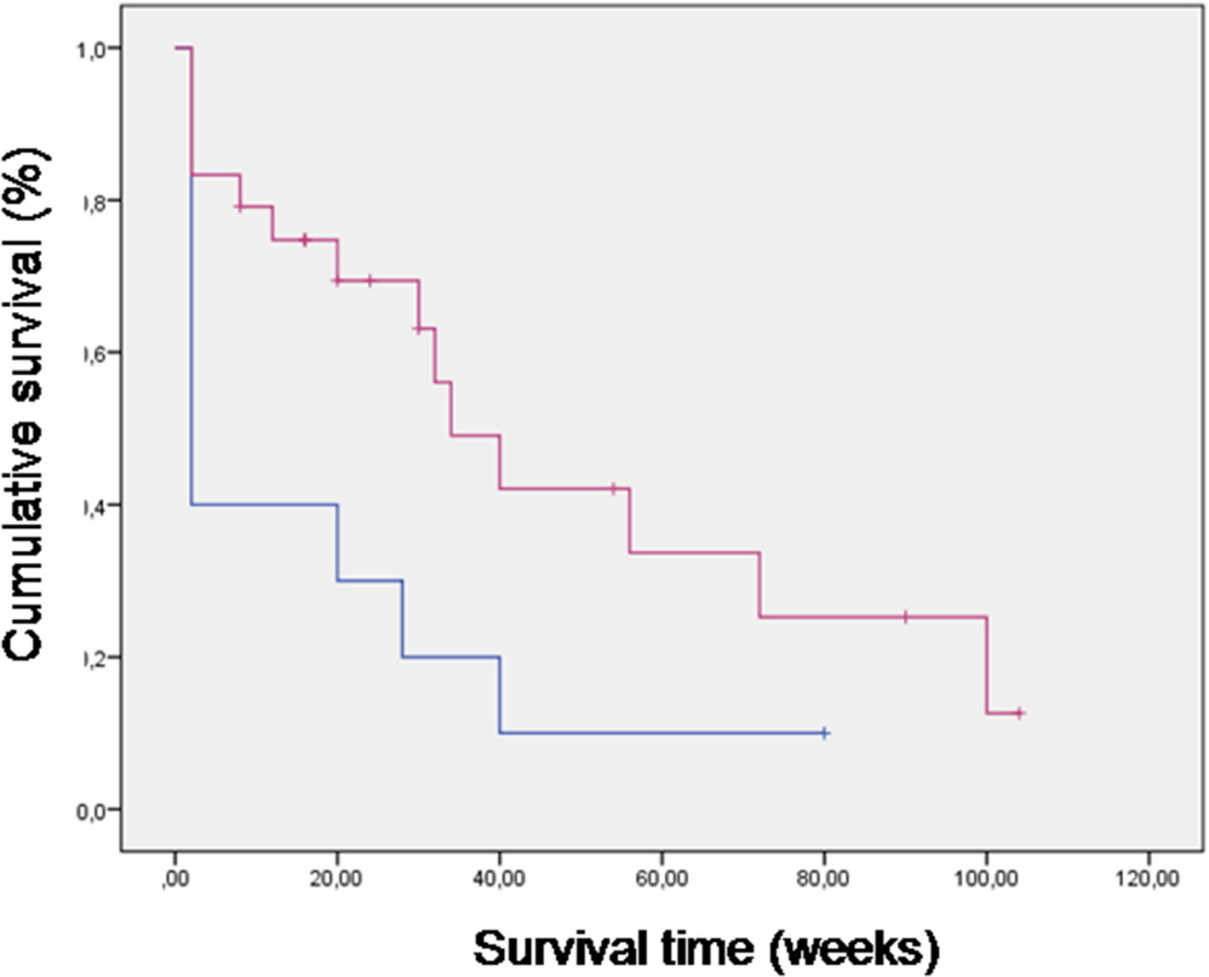
**Kaplan-Meier survival curve for alkalized patients (red line) and controls (blue line).**

**Figure 4:**
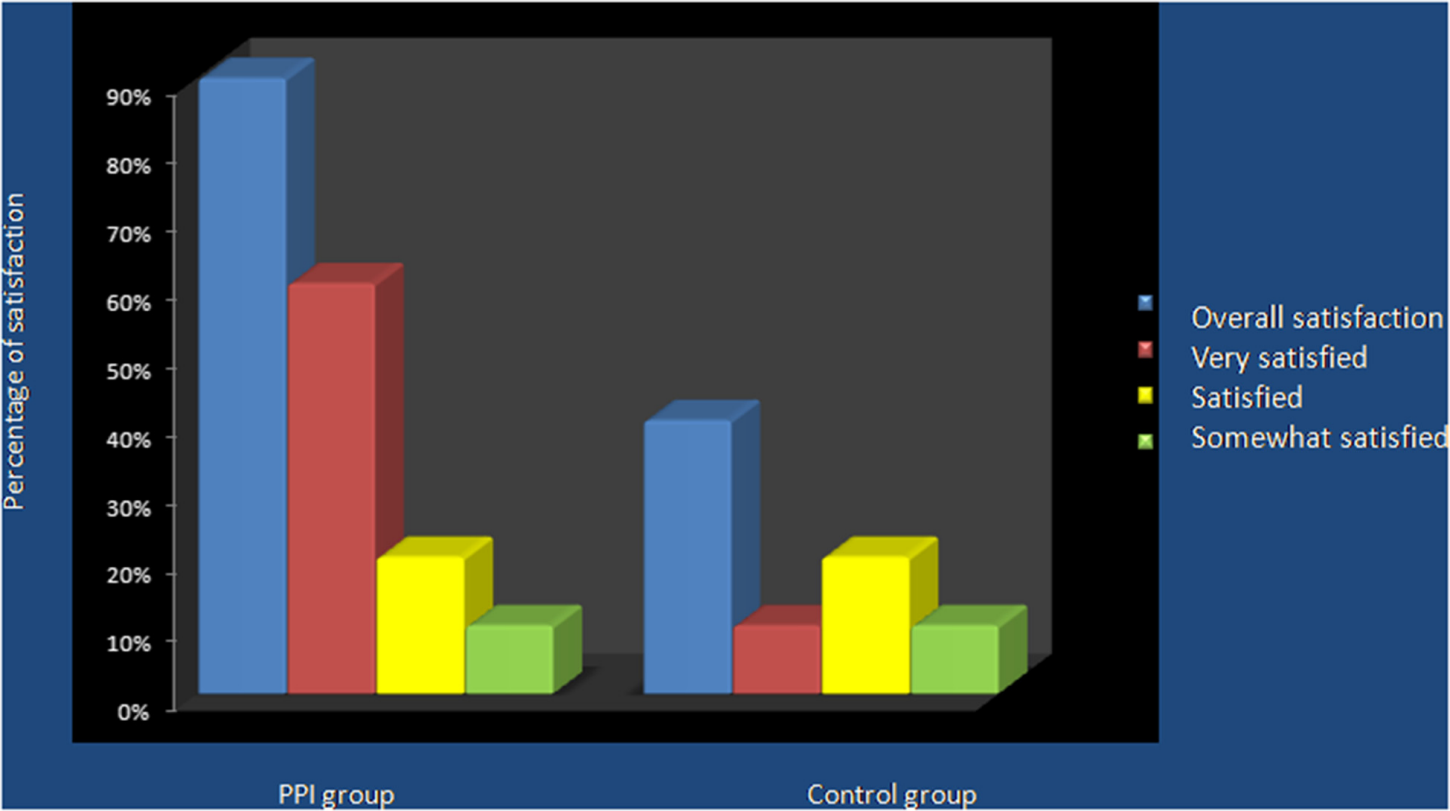
**Histogram representation of the owners’ percentage and degree of satisfaction for the clinical outcome of their pets in the PPI and control groups.**

Twenty-four pets affected by different solid tumors were enrolled in the study (twenty-two dogs and two cats) over a 30 months period. There were 11 male dogs and 11 female dogs, most of whom where spayed while the two cats were both female. Age ranged from 6 to 13 years. Patients characteristics and response to therapy are shown in Table 4. Eight patient had previous surgery for their primary tumors (six of them had either regional metastases or local recurrence when referred for therapy) and one had three sessions of electrochemotherapy resulting in partial remission of the tumor. This last patient then switched to metronomic chemotherapy for financial reasons and further improved the tumor response. None of the patients with metastatic disease had metastasectomy performed prior to referral, having all the owners elected their pets to be pharmacologically treated. The only patients that has no evidence of disease at the time of referral were four patients with ruptured splenic hemangiosarcoma (two per each cohort) that have been enrolled in the survival study in consideration of the extremely high metastatic tendency of this neoplasm in the canine population. All the four patients begun their medical therapy two weeks after splenectomy. Survival time was calculated from the beginning of metronomic chemotherapy. The historical control group consisted with 10 dogs affected by different neoplasms whose characteristics are summarized in Table 5. In this group there were 6 females and 4 males who tolerated the metronomic therapy without side effects. The combination of metronomic chemotherapy and alkalizing treatment was overall well tolerated, one dog had mild diarrhea but continued the therapy albeit at a decreased lansoprazole dose (2 mg/kg in the three loading days). Two dogs had vomiting that resulted in lansoprazole reduction from 5 mg/kg to 3 mg/kg. Finally 8 dogs out of 22 experienced different degrees of flatulence that partially improved with the addition of probiotics to their diet. The overall responses rate was 75%, including 4 complete remissions, 10 partial responses, 4 no evidence of disease (adjuvant therapy group), 2 stable diseases and 4 progressive diseases. On the other hand, the overall response rate in the control group was 40% with two short lived NEDs in dogs with highly metastatic cancers, one PR in a dog with a ulnar OSA that recurred after partial ulnectomy and one long lasting CR in a dog with an inflammatory mammary carcinoma. In this cohort only one patient out of ten experienced a complete long lasting response, while all the other were non responders or short lived responders. Figure 1 shows two patients that successfully responded to the therapy while Figure 2 shows a stable disease in a dog with a large lung tumor. At the time of writing a total of 9 patients (8 dogs and 1 cat) are still alive and periodically monitored leading to a survival rate of 37.5% while only one control patient is still alive with a survival rate of 10%. The mean time to terminal event was 48 weeks in the experimental cohort and 18 weeks in the control group, median time to terminal event was 34 weeks versus 2. Figure 3 shows the Kaplan-Meier survavial curve for the two groups. In general, the owners of the experimental group reported increased activity level as well as food and water consumption and improved quality of life. Questioning the owners regarding their degree of satisfaction with the outcome of the therapy yielded a total of 90% of appreciation in the PPI group and a 40% in the control group. Appreciation was greatly influenced on the pets moving up in the karnofsky scale as a consequence of improved response and degree of side effects and ranged from “somewhat satisfied” to “enthusiastic”. Figure 4 summarizes the degree of satisfaction among the different groups of owners. While tumor control was the major issue in the control group, in the PPI cohort, as a consequence of a better clinical outcome, the major causes of complains were the gastrointestinal side effects (specifically the flatulence) experienced by some dogs rather than the degree of tumor response. The management of these complications through diet and integration with probiotics greatly improved the owners degree of satisfaction.

**Table 4:** **Patients characteristics and outcome of 24 pets with advanced cancer treated with metronomic chemotherapy and alkalization**

**Patient**	**Age**	**Sex**	**Tumor**	**Prev. treatment**	**Tumor stage**	**Outcome (weeks)**
Mixed breed	10	FS	Metastatic Anal sac Ca (sublumbar lymph nodes)	Surgery	T_0_ N_1_ M_X_	PR 40
Mixed breed	12	FS	Metastatic Anal sac Ca (sublumbar lymph nodes)	Surgery	T_0_ N_1_ M_X_	CR 56
Mixed breed	16	FS	Thyroid Ca	Biopsy	T_3B_ N_0_ M_X_	PD
Cocker Spaniel	6	MC	Liver Sarcoma	Biopsy	T_2_ N_0_ M_X_	PR 30
Dachshund	13	MC	Liver Ca	Biopsy	T_2_ N_0_ M_X_	CR 90+
Golden retriever	14	MC	Ruptured	Surgery	T_3_ N_0_ M_X_	NED 72
			Splenic HSA			
Labrador retriever	12	MC	Ruptured	Surgery	T_3_ N_0_ M_X_	NED 34
			Splenic HSA			
Golden retriever	13	FS	Recurring inflammatory mammary Ca	Surgery	T_4_ N_0_ M_X_	CR 104+
Golden retriever	10	FS	Recurring inflammatory mammary Ca	Surgery	T_4_ N_0_ M_X_	PD
Boxer	10	FS	Metastatic mammary Ca (axillary lymph node)	Biopsy	T_3_ N_1_ M_X_	PR 32
Dachshund	13	MC	Limb Sarcoma	Biopsy	T_3_ N_0_ M_X_	CR 20
Labrador retriever	11	M	Recurring	Surgery	T_2_ N_0_ M_X_	NED 30
			Neck Sarcoma			
Mixed breed	13	FS	Nasal sinus AdCa	Biopsy	T_3_ N_0_ M_X_	PR 24+
Labrador	10	FS	Nasal Sarcoma	Electrochemotherapy	T_3_ N_0_ M_X_	PR 20+
Tibetan spaniel	11	M	Nasal carcinoma	Biopsy	T_3_ N_0_ M_X_	PR 8
Visla	10	M	Nasal carcinoma	Biopsy	T_2_ N_0_ M_X_	PR16+
Mixed breed	10	F	Lingual SCC	Biopsy	T_2_ N_1A_ M_X_	PR 16+
Mixed breed	12	FS	Lung carcinoma	Biopsy	T_1_ N_X_ M_X_	SD 16+
Mixed breed	13	F	Lung carcinoma	Biopsy	T_1_ N_X_ M_X_	PR 12
German Shepherd	10	M	Visceral histiocytosis	Biopsy	T_3_ N_1_ M_X_	PD
German shepherd	9	M	OSA	Biopsy	T_2_ N_X_ M_X_	PD
Rottweiler	8	M	OSA	Biopsy	T_2_ N_X_ M_X_	SD 8+
DSH	10	FS	Nasal CSA	Biopsy	T_2_ N_0_ M_X_	PR 100
DSH	10	FS	Recurring anaplastic mammary carcinoma	Surgery	T_4_ N_0_ M_X_	NED 54+

The tumor stage refers at the moment of referral. HSA patients were referred after the surgical removal of their ruptured spleen and were the only patients without gross disease.

*Abbreviations*: *CA* carcinoma, *CR* complete remission, *CSA* chondrosarcoma, *F* female, *FS* female spayed, *M* male, *MC* male castrated, *NED* no evidence of disease, *OSA* osteosarcoma, *PD* progressive disease, *PR* partial remission, *SCC* squamous cell carcinoma, *SD* stable disease.

**Table 5:** **Patients characteristics and outcome of 10 pets with advanced cancer treated with metronomic chemotherapy alone**

**Patient**	**Age**	**Sex**	**Tumor**	**Prev. treatment**	**Tumor stage**	**Outcome (weeks)**
Mixed breed	13	FS	Metastatic	Surgery	T_0_ N_1_ M_X_	PD
			Anal sac Ca (sublumbar lymph nodes)			
Poodle	12	MC	Thyroid Ca	Biopsy	T_3B_ N_0_ M_X_	PD
Labrador	9	F	Liver CA	Biopsy	T_2_ N_0_ M_X_	PD
Golden retriever	11	MC	Ruptured	Surgery	T_3_ N_0_ M_X_	NED 28
			Splenic HSA			
German Shepherd	10	MC	Ruptured	Surgery	T_3_ N_0_ M_X_	NED 20
			Splenic HSA			
Boxer	11	FS	Inflammatory mammary Ca	Biopsy	T_4_ N_0_ M_X_	CR 80+
Mixed breed	13	FS	Recurring	Surgery	T_4_ N_0_ M_X_	PD
			Inflammatory mammary Ca			
Boxer	10	FS	Mammary Ca	Biopsy	T_3C_ N_1_ M_X_	PD
Doberman	13	FS	Anaplastic mammary Ca (axillary lymph node)	Biopsy	T_3B_ N_1_ M_X_	PD
Boxer	11	M C	OSA	Biopsy	T_2_ N_X_ M_X_	PR 40

*Abbreviations*: *CA* carcinoma, *CR* complete remission, *F* female, *FS* female spayed, *M* male, *MC* male castrated, *NED* no evidence of disease, *OSA* osteosarcoma, *PD* progressive disease, *PR* partial remission.

## Conclusions

The proton pump inhibitor lansoprazole, administered at high dose and combined with a water alkalizer, has proven to enhance tumor response to metronomic chemotherapy, increasing the number of complete responders [4] and significantly delaying the onset of metastases in pets with highly metastasizing neoplasms. This is a very promising result, since a “flaw” of metronomic chemotherapy is the low number of patients experiencing complete responses as well as the difficulty to have a significant delay for the onset of metastatic disease in patients with advanced cancer [10-17]. The broad spectrum of solid tumors that responded to this clinical approach including two patients with metastasized anal sac carcinoma makes this combination extremely promising. Of course this study had some limitations due to the lack of tumor homogeneity in our two cohorts. This bias can’t be overcome, inasmuch pets with advanced cancer stage suitable to be included to this study presented tumors that very often varied in their histologies. Moreover, the owners of the pets affected with advanced cancer were particularly available in trying new approached being well aware of the disease in their companion animals and of their expected poor prognosis. Notably, although the level of cancer progression and the size of the cancer, we observed a clear improvement of the clinical response to the metronomic therapy when combined with an alkalinizing treatment, independently from the nature of cancer, suggesting that systemic alkalinisation might represent one of the most important new implementation in cancer therapy, inasmuch as tumor extracellular acidity is a feature common to all cancers. This is for sure a novelty in the clinical management of cancer patients that should be taken into careful account in the future anti-cancer strategies. Of course our study lacked a system to assess the in vivo pH changes as well; this was due to the absence of pH measurement methodology approved for the clinical use but also to the financial constrains to an observational setting. In order to improve the protocol and to identify prognostic factors, steps should be made to measure changes in tumor pH during the therapy in order to monitor progresses and also to have a parameter to be monitored in order to identify escape from medical control. Furthermore, a reliable and portable system to quantify variation in growth factors involved in tumor progression and angiogenesis should be devised [17]. The combination of water alkalinisation and high dosage proton pump inhibitors has been shown to effectiveness of metronomic therapy on tumour progression, but also to highly improve the quality of life of pets affected by spontaneous malignant tumors. In particular the animals did not show to refuse the alkalinized water, while occasional experience in human patients showed that sodium bicarbonate may not be administered for a long period of time, inasmuch as patients very often refused it. Notably, while this study allowed to try with a real experimental clinical approach against advanced cancer, it did not allow to quantify the exact contribution of PPIs and alkalized water to the increased efficacy of metronomic chemotherapy, while alkalinizing approaches using sodium bicarbonate suggest a potential efficacy of water alkalinisation in cancer treatment [33]. Further studies will be mandatory to evaluate the effects of single alkalinizing approaches as potential anti-cancer treatments . In our study the owners of the pet included in the experimental group provided the emotional component and a daily evaluation of the quality of life, providing a rough estimate of the patient toleration of the therapeutic regimen, allowing the investigators to work in a close approximation of the human clinical condition. Moreover, sodium bicarbonate is an unbalanced buffer with only sodium as an oligoelement, and this may raise cardiovascular problems in long term administrations. The inclusion of multiple drugs affecting tumor acidity is highly desirable in order to develop multidrug protocols to increase cancer control [33]. Finally, there is accumulating evidence also indicating that some metronomic regimens might be able to promote disease eradication by stimulating anticancer immune and selectively eliminating immunosuppressive cells [9,15]. The increased anti-tumor immune response by proton pump inhibitors has been clearly shown [34,35], and the overall response to the combination of proton pump inhibitors, alkalinized water and metronomic therapy, suggest that this approach may be highly helpful in the generalized response of the body against cancer. Further investigations are clearly warranted also in this perspective to assess the weight of the immune component in the long term control of tumors in patient undergoing alkalizing metronomic chemotherapy [35].

## References

[1] Chen Z, Cui Y, Owonikoko TK, Wang Z, Li Z, Luo R, Kutner M, Khuri FR, Kowalski J: **Escalation with overdose control using all toxicities and time to event toxicity data in cancer phase I clinical trials.** *Contemp Clin Trials* 2014, Epub ahead of print. 10.1016/j.cct.2014.02.004 PMC4046505 24530487

[2] Jang SH, Wientjes MG, Lu D, Au JLS (2003). Drug delivery and transport to solid tumors. Pharm Res.

[3] Kim JJ, Tannock IF (2005). Repopulation of cancer cells during therapy: an important cause of treatment failure. Nat Rev Cancer.

[4] Davis AJ, Tannock JF (2000). Repopulation of tumour cells between cycles of chemotherapy: a neglected factor. Lancet Oncol.

[5] Kerbel RS, Kamen BA (2004). The anti-angiogenic basis of metronomic chemotherapy. Nat Rev Cancer.

[6] Gasparini G (2001). Metronomic scheduling: the future of chemotherapy?. Lancet Oncol.

[7] Mross K, Steinbild S (2012). Metronomic anti-cancer therapy – an ongoing treatment option for advanced cancer patients. J Canc Res Therapeut.

[8] Browder T, Butterfield CE, Kräling BM, Shi B, Marshall B, O’Reilly MS, Folkman J (2000). Antiangiogenic scheduling of chemotherapy improves efficacy against experimental drug-resistant cancer. Cancer Res.

[9] Sheng Sow H, Mattarollo SR (2013). Combining low-dose metronomic chemotherapy with anticancer vaccines: a therapeutic opportunity for lymphomas. Oncoimmunology.

[10] Adenis A, Ray-Coquard I, Italiano A, Chauzit E, Bui-Nguyen B, Blay JY, Tresch-Bruneel E, Fournier C, Clisant S, Amela EY, Cassier PA, Molimard M, Penel N (2013). A dose-escalating phase I of imatinib mesylate with fixed dose of metronomic cyclophosphamide in targeted solid tumours. Br J Cancer.

[11] Robison NJ, Campigotto F, Chi SN, Manley PE, Turner CD, Zimmerman MA, Chordas CA, Werger AM, Allen JC, Goldman S, Rubin JB, Isakoff MS, Pan WJ, Khatib ZA, Comito MA, Bendel AE, Pietrantonio JB, Kondrat L, Hubbs SM, Neuberg DS, Kieran MW (2014). A phase II trial of a multi-agent oral antiangiogenic (metronomic) regimen in children with recurrent or progressive cancer. Pediatr Blood Cancer.

[12] Lana S, U’ren L, Plaza S, Elmslie R, Gustafson D, Morley P, Dow S (2007). Continuous low-dose oral chemotherapy for adjuvant therapy of splenic hemangiosarcoma in dogs. J Vet Intern Med.

[13] Leach TN, Childress MO, Greene SN, Mohamed AS, Moore GE, Schrempp DR, Lahrman SR, Knapp DW (2012). Prospective trial of metronomic chlorambucil chemotherapy in dogs with naturally occurring cancer. Vet Comp Oncol.

[14] Schrempp DR, Childress MO, Stewart JC, Leach TN, Tan KM, Abbo AH, de Gortari AE, Bonney PL, Knapp DW (2013). Metronomic administration of chlorambucil for treatment of dogs with urinary bladder transitional cell carcinoma. J Am Vet Med Assoc.

[15] Mitchell L, Thamm DH, Biller BJ (2012). Clinical and immunomodulatory effects of toceranib combined with low-dose cyclophosphamide in dogs with cancer. J Vet Intern Med.

[16] Elmslie RE, Glawe P, Dow SW (2008). Metronomic therapy with cyclophosphamide and piroxicam effectively delays tumor recurrence in dogs with incompletely resected soft tissue sarcomas. J Vet Intern Med.

[17] Marchetti V, Giorgi M, Fioravanti A, Finotello R, Citi S, Canu B, Orlandi P, Di Desidero T, Danesi R, Bocci G (2012). First-line metronomic chemotherapy in a metastatic model of spontaneous canine tumours: a pilot study. Invest New Drugs.

[18] De Milito A, Fais S (2005). Tumor acidity, chemoresistance and proton pump inhibitors. Future Oncol.

[19] Fais S (2010). Proton pump inhibitor-induced tumour cell death by inhibition of a detoxification mechanism. J Intern Med.

[20] Cipriano DJ, Wang Y, Bond S, Hinton A, Jefferies KC, Qi J, Forgac M (2008). Structure and regulation of the vacuolar ATPases. Biochim Biophys Acta.

[21] Jefferies KC, Cipriano DJ, Forgac M (2008). Function, structure and regulation of the vacuolar (H+)-ATPases. Arch Biochem Biophys.

[22] Spugnini EP, Citro G, Fais S (2010). Proton pump inhibitors as anti vacuolar-ATPases drugs: a novel anticancer strategy. J Exp Clin Cancer Res.

[23] Murakami T, Shibuya I, Ise T, Chen ZS, Akiyama S, Nakagawa M, Izumi H, Nakamura T, Matsuo K, Yamada Y, Kohno K (2001). Elevated expression of vacuolar proton pump genes and cellular PH in cisplatin resistance. Int J Cancer.

[24] De Milito A, Iessi E, Logozzi M, Lozupone F, Spada M, Marino ML, Federici C, Perdicchio M, Matarrese P, Lugini L, Nilsson A, Fais S (2007). Proton pump inhibitors induce apoptosis of human B-cell tumors through a caspase-independent mechanism involving reactive oxygen species. Cancer Res.

[25] Federici C, Petrucci F, Caimi S, Cesolini A, Logozzi M, Borghi M, D’Ilio S, Lugini L, Violante N, Azzarito T, Majorani C, Brambilla D, Fais S (2014). Exosome release and low pH belong to a framework of resistance of human melanoma cells to cisplatin. PLoS One.

[26] Spugnini EP, Baldi A, Buglioni S, Carocci F, Milesi de Bazzichini G, Betti G, Pantaleo I, Menicagli F, Citro G, Fais S (2011). Lansoprazole as a rescue agent in chemoresistant tumors: a phase I/II study in companion animals with spontaneously occurring tumors. J Transl Med.

[27] Ferrari S, Perut F, Fagioli F, Brach Del Prever A, Meazza C, Parafioriti A, Picci P, Gambarotti M, Avnet S, Baldini N, Fais S (2013). Proton pump inhibitor chemosensitization in human osteosarcoma: from the bench to the patients’ bed. J Transl Med.

[28] Ibrahim-Hashim A, Cornnell HH, Abrahams D, Lloyd M, Bui M, Gillies RJ, Gatenby RA (2012). Systemic buffers inhibit carcinogenesis in TRAMP mice. J Urol.

[29] Robey IF, Baggett BK, Kirkpatrick ND, Roe DJ, Dosescu J, Sloane BF, Hashim AI, Morse DL, Raghunand N, Gatenby RA, Gillies RJ (2009). Bicarbonate increases tumor pH and inhibits spontaneous metastases. Cancer Res.

[30] Chretin JD, Rassnick KM, Shaw NA, Hahn KA, Ogilvie GK, Kristal O, Northrup NC, Moore AS (2007). Prophylactic trimethoprim-sulfadiazine during chemotherapy in dogs with lymphoma and osteosarcoma: a double-blind, placebo-controlled study. J Vet Intern Med.

[31] Kaplan EL, Meier P (1958). Nonparametric estimation from incomplete observations. J Am Stat Assoc.

[32] Peto R, Pike MC, Armitage P, Breslow NE, Cox DR, Howard SV, Mantel N, McPherson K, Peto J, Smith PG (1977). Design and analysis of randomized clinical trials requiring prolonged observation of each patient: II analysis and examples. Br J Cancer.

[33] Robey IF, Martin NK (2011). Bicarbonate and dichloroacetate: evaluating pH altering therapies in a mouse model for metastatic breast cancer. BMC Cancer.

[34] Huber V, De Milito A, Harguindey S, Reshkin SJ, Wahl ML, Rauch C, Chiesi A, Pouysségur J, Gatenby RA, Rivoltini L, Fais S (2010). Proton dynamics in cancer. J Transl Med.

[35] Calcinotto A, Filipazzi P, Grioni M, Iero M, De Milito A, Ricupito A, Cova A, Canese R, Jachetti E, Rossetti M, Huber V, Parmiani G, Generoso L, Santinami M, Borghi M, Fais S, Bellone M, Rivoltini L (2012). Modulation of microenvironment acidity reverses anergy in human and murine tumor-infiltrating T lymphocytes. Cancer Res.

